# The Effect of Intravenous Lidocaine Treatment on Sleep and Quality of Life in Fibromyalgia: An Observational Study

**DOI:** 10.3390/jcm15082887

**Published:** 2026-04-10

**Authors:** Halil Ibrahim Altun, Fatma Aysen Eren

**Affiliations:** Department of Anesthesiology, Division of Pain Medicine, Istanbul Kanuni Sultan Suleyman Training and Research Hospital, Istanbul 34303, Turkey

**Keywords:** fibromyalgia, intravenous lidocaine, sleep quality, quality of life, pain

## Abstract

**Background/Objectives**: Fibromyalgia is a painful syndrome with biopsychosocial components that predominantly affects middle-aged women. This study aimed to evaluate changes in sleep quality and quality of life following intravenous (IV) lidocaine treatment in patients with fibromyalgia (FM). **Methods**: This retrospective observational study included patients diagnosed with fibromyalgia who underwent intravenous lidocaine treatment at a tertiary pain clinic between June 2023 and June 2024 and had a Pittsburgh Sleep Quality Index (PSQI) score > 5. The patients’ demographic data, Fibromyalgia Impact Questionnaire (FIQ) scores at baseline and at 1 and 3 months post-treatment, Numerical Rating Scale (NRS-11) scores, Short Form-12 (SF-12) mental and physical component scores (MCS-12, PCS-12), and PSQI scores were recorded. **Results**: Overall, 51 patients were included. 92.2% of the patients were women, with a mean age of 41.6 ± 9.5 years. Statistically significant reductions in NRS-11, FIQ, and PSQI scores and increases in SF-12 component scores were observed at 1 and 3 months compared with baseline (*p* < 0.001). Negative correlations were found between NRS-11 and PCS-12 and MCS-12, and a positive correlation was found between FIQ and PSQI. Sleep quality showed a marked improvement at 1 month; however, attenuation of this benefit was observed at the 3-month follow-up. **Conclusions**: Sleep quality appeared to be associated with short-term functional outcomes, whereas pain intensity was associated with mid-term clinical status in patients with fibromyalgia. Prospective randomized controlled trials are required to confirm these findings and to determine optimal dosing and treatment schedules.

## 1. Introduction

Fibromyalgia (FM) is a chronic condition involving a complex interaction of biological, psychological, and social factors, and it significantly affects patients’ quality of life. Patients commonly experience diffuse musculoskeletal pain accompanied by sleep disturbances, fatigue, and morning stiffness, although the intensity and constellation of these symptoms can differ among individuals. While FM can occur at any age, it is most frequently seen in middle-aged women and is estimated to have a prevalence of approximately 2% within the general population. The exact pathophysiological mechanisms remain unclear; however, current evidence suggests that genetic susceptibility, central and peripheral sensitization, and dysfunction of the autonomic nervous system may contribute to disease development [1,2]. In addition, alterations in central neurotransmitter levels, including serotonin, dopamine, and norepinephrine, have been reported in individuals with FM, which may further explain the complexity of symptom presentation [3].

The clinical presentation of fibromyalgia is highly heterogeneous, with symptoms varying considerably between individuals. Pain may initially be localized but often becomes more widespread over time. On physical examination, patients frequently exhibit features such as allodynia and hyperalgesia. In addition to pain, a wide range of accompanying conditions may be observed, including irritable bowel syndrome, mood disorders (e.g., depression), primary headache disorders, restless leg syndrome, vasomotor disturbances like Raynaud’s phenomenon, and various autonomic or somatic complaints such as palpitations, dry mouth, and fatigue. Other manifestations, such as sexual dysfunction, dysmenorrhea, and anxiety disorders, may also be present [4,5]. Although these comorbid conditions are important in the differential diagnosis, their presence does not exclude fibromyalgia. In clinical practice, diagnosis is commonly based on the revised 2016 American College of Rheumatology (ACR) criteria [6].

Managing fibromyalgia remains challenging and often requires a flexible, patient-centered, multimodal approach. Non-pharmacological strategies—such as lifestyle adjustments, regular exercise, and mind–body practices like meditation and yoga—are generally considered key components of care. Although medications, particularly antidepressants, are frequently prescribed, their benefits are not always consistent and may be limited by side effects and poor adherence. In this setting, complementary and interventional techniques, including acupuncture, dry needling, and trigger point injections, may provide additional relief for some patients [7,8]. Given the wide variability in symptom severity and pain experience, tailoring treatment to the individual patient is essential for achieving meaningful clinical improvement.

Intravenous (IV) therapies have increasingly been used in the management of chronic pain, mainly because of their potential to influence both peripheral and central sensitization. In everyday clinical practice, they are often combined with standard medical and interventional treatments. Magnesium, lidocaine, and ketamine are among the most commonly used agents. However, there is still no clear consensus regarding their optimal dosing or treatment intervals. Although evidence on the use of IV lidocaine in fibromyalgia is still limited, it has already found a place in clinical practice for selected patients [9,10].

Sleep disturbances and reduced quality of life are common in patients with fibromyalgia and often contribute to the overall symptom burden [11]. While intravenous lidocaine has been reported to improve certain symptoms in FM, its effects on sleep quality and quality of life have not been clearly established. In particular, there is a lack of focused evidence addressing these outcomes. Although previous studies have primarily focused on pain-related outcomes following intravenous lidocaine treatment in fibromyalgia, data regarding its effects on sleep quality and health-related quality of life remain limited. In addition, the relative contributions of pain and sleep domains to clinical outcomes over time have not been clearly explored [7,9,10]. For this reason, the present study aimed to explore the relationship between intravenous lidocaine treatment and changes in sleep quality and quality of life in patients with fibromyalgia, as well as how these outcomes evolve over time in a real-world clinical setting.

## 2. Materials and Methods

This research was conducted as a retrospective, single-center observational cohort study in a tertiary pain management setting. We reviewed the medical records of patients diagnosed with fibromyalgia (FM) according to the 2016 American College of Rheumatology (ACR) criteria who received intravenous (IV) lidocaine treatment between June 2023 and June 2024.

Ethical approval was obtained from the Institutional Ethics Committee (Approval No. KAEK/2024.10.212). All patients had previously provided written informed consent for the use of their clinical data. Given the retrospective nature of the study, additional consent was not required. The study was also registered at ClinicalTrials.gov (Identifier: NCT07100665) and conducted in accordance with the Declaration of Helsinki.

Eligible participants were adults aged between 18 and 65 years who fulfilled the 2016 ACR diagnostic criteria for fibromyalgia and received intravenous lidocaine during the study period. To ensure a more homogeneous sample, we included patients with persistent pain despite ongoing treatment (NRS-11 > 4) and impaired sleep quality (PSQI > 5). Only those who completed the treatment protocol and had complete baseline and follow-up data were analyzed.

Patients were excluded if their fibromyalgia-related treatments were changed during the follow-up period or if clinical data were incomplete. Additional exclusion criteria included significant cardiovascular disease, renal or hepatic failure, uncontrolled metabolic disorders, pregnancy, active infection or malignancy, neuromuscular disease, psychiatric disorders, known hypersensitivity to lidocaine, abnormal electrolyte levels, and the use of magnesium or vitamin D supplementation.

Demographic and clinical data—including age, sex, body mass index (BMI), symptom duration, comorbidities, and current treatments—were obtained from medical records. Disease impact was assessed using the Fibromyalgia Impact Questionnaire (FIQ), pain intensity with the Numerical Rating Scale (NRS-11), and health-related quality of life with the Short Form-12 (SF-12), including both the mental (MCS-12) and physical (PCS-12) component scores. Sleep quality was evaluated using the Pittsburgh Sleep Quality Index (PSQI).

All outcome measures were recorded at baseline, prior to the first infusion, and reassessed at 1 and 3 months after treatment. The study was intended to provide real-world observational data rather than to establish causal treatment effects.

Adverse events were systematically recorded during each infusion session and the post-infusion observation period based on patient-reported symptoms and clinical observations and documented in the medical records. These data were obtained from routinely recorded clinical documentation.

This study was conducted and reported in line with the Strengthening the Reporting of Observational Studies in Epidemiology (STROBE) guidelines. The completed STROBE checklist is provided as a Supplementary Materials File [12,13].

### 2.1. Procedure

Patients were monitored in a dedicated procedure room using continuous electrocardiography, pulse oximetry, and non-invasive blood pressure measurements. Intravenous access was established in a forearm vein before the procedure. Before starting the infusion, a 12-lead electrocardiogram was obtained to exclude underlying arrhythmias, and patients were informed about potential adverse effects.

The IV lidocaine protocol consisted of a dose of 5 mg/kg diluted in 0.9% saline to a total volume of 250 mL. The infusion was administered over 2 h at a rate of 125 mL/h using an infusion pump, under direct medical supervision. Serum lidocaine levels were not routinely measured; however, all patients were continuously monitored during infusion. Treatment was performed once weekly for a total of five sessions, in line with current clinical practice [9,10,11]. After each session, patients were observed for an additional 2 h for possible adverse effects.

### 2.2. Assessment Scales

The original Fibromyalgia Impact Questionnaire (FIQ) evaluates disease burden on a scale from 0 to 100, with higher scores reflecting greater impact [14].

Pain severity was assessed using the Numerical Rating Scale (NRS-11), where patients rate their pain intensity on a scale from 0 (no pain) to 10 (worst imaginable pain) [15].

The Short Form-12 (SF-12) evaluates health-related quality of life across physical and mental domains. The Physical Component Score (PCS-12) and Mental Component Score (MCS-12) range from 0 to 100, with higher scores indicating better health status [16].

Sleep quality was measured using the Pittsburgh Sleep Quality Index (PSQI), a validated tool in which higher scores indicate poorer sleep quality, with values above 5 suggesting clinically significant impairment [17].

### 2.3. Statistical Analysis

Data were analyzed using IBM SPSS v23 and IBM AMOS v24 (IBM Corp., Armonk, NY, USA). Descriptive statistics were presented as the number of units (n), percentages (%) for categorical variables and as means ± standard deviations, medians, and minimum and maximum for continuous variables, as appropriate. Conformity of the data to normal distribution was examined using the Kolmogorov–Smirnov test. The Friedman test was used to analyze median NRS-11, FIQ, SF-12, and PSQI scores during the follow-up period, provided that the distributions were not normal. When a significant overall difference was detected, pairwise comparisons were performed using post hoc Wilcoxon signed-rank tests, with *p*-values adjusted by the Bonferroni correction for multiple comparisons. The relationship between quantitative variables with normal distributions was analyzed using Pearson’s correlation. The relationship between non-normally distributed quantitative variables was examined using Spearman’s rho correlation. Path analysis was used to test structural models. In the path analysis examining the t0–t1 change, the NRS-11 scale was not included because it caused multiple multicollinearity problems in the dataset.

### 2.4. Sample Size

The sample size was calculated using G*Power software (version 3.1.9.4). Based on a non-parametric analytical approach, an effect size of 0.5, a statistical power of 0.95, and a significance level of 0.05 were assumed. Based on these assumptions, the minimum required sample size was calculated as 47 participants. A total of 51 patients were ultimately included, slightly exceeding this threshold and allowing for adequate statistical power for the planned analyses, including path modeling.

## 3. Results

**Figure 1:**
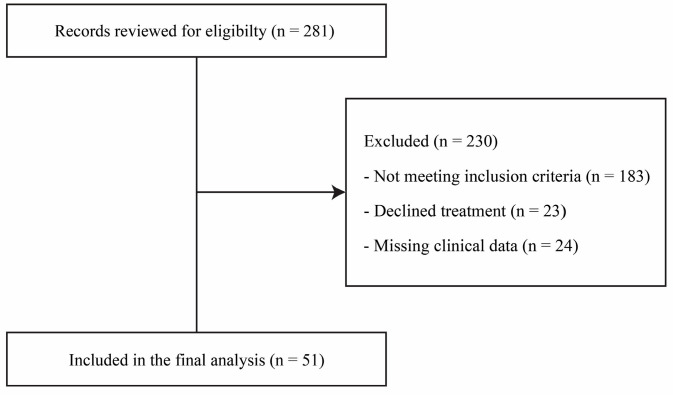
Flowchart.

A total of 51 patients were analyzed (Figure 1), the majority of whom were female (92.2%), with a mean age of 41.6 ± 9.5 years.

The mean body mass index (BMI) was 27.0 ± 3.7 kg/m^2^, and the average duration of symptoms was 4.3 ± 3.9 years. Comorbid conditions were present in 13.7% of patients. All patients were receiving ongoing pharmacological treatment for fibromyalgia, as shown below (Table 1).

Significant changes were observed in all outcome measures over time. Compared with baseline, NRS-11, FIQ, and PSQI scores showed a marked reduction at both 1 and 3 months, while SF-12 physical and mental component scores increased significantly (*p* < 0.001 for all comparisons) (Table 2).

A responder analysis based on NRS-11 showed that 23.5% of patients achieved ≥30% pain reduction and 15.7% achieved ≥50% reduction. However, 56.9% were classified as non-responders, and 3.9% showed worsening (Table 3). At 3 months, 78.4% of patients remained above the PSQI threshold (>5), while only 21.6% achieved good sleep quality (Table 4).

Correlation analyses demonstrated a strong negative relationship between pain intensity (NRS-11) and both physical and mental quality of life (PCS-12 and MCS-12) at 1 month (r = −0.87 and r = −0.91, respectively; *p* < 0.001). In contrast, positive correlations were observed between NRS-11 and FIQ (r = 0.78, *p* < 0.001) as well as PSQI scores (r = 0.72, *p* < 0.001), indicating that higher pain levels were associated with greater disease burden and poorer sleep quality (Table 5).

At the 3-month follow-up, similar patterns were observed. Pain intensity remained negatively correlated with quality of life measures and positively correlated with disease impact and sleep disturbance, although the strength of these associations was slightly reduced compared to the 1-month results. (Table 6).

Path analysis examining short-term changes (baseline to 1 month) indicated that sleep quality was significantly associated with both physical and mental components of quality of life as well as disease impact. Due to multicollinearity, pain intensity (NRS-11) was not included in this model. These findings indicate a stronger association between sleep-related factors and early post-treatment outcomes (Table 7).

In contrast, path analysis of longer-term changes (baseline to 3 months) did not show a significant association between sleep quality and functional outcomes. Instead, pain intensity emerged as the primary factor associated with physical functioning, mental well-being, and overall disease impact (Table 8).

Regarding safety, no serious adverse events were observed during the study period. Minor side effects were reported in a small number of patients, including transient tachycardia (*n* = 4), tongue numbness (*n* = 1), and tinnitus (*n* = 2). All adverse events were self-limiting and did not require additional intervention. No patients discontinued treatment due to adverse events.

Overall, the greatest improvement was observed at 1 month, with a partial reduction in effect at 3 months.

## 4. Discussion

In this observational study, improvements in clinical outcomes were observed at both 1 and 3 months following intravenous lidocaine treatment. Although these changes reached statistical significance, responder analysis indicated that clinically meaningful pain reduction was achieved in only a subset of patients. In fact, just under a quarter of patients experienced a ≥30% reduction in pain, and an even smaller proportion reached a ≥50% response, while more than half were classified as non-responders and a few reported worsening symptoms. Taken together, this pattern suggests that the overall treatment effect is modest and varies considerably between individuals. The demographic characteristics of our cohort, predominantly middle-aged women, were consistent with the typical profile of fibromyalgia reported in the literature [18].

Pharmacological treatment remains an important part of fibromyalgia management, although achieving consistent symptom control can be difficult in everyday clinical practice. In our cohort, a notable proportion of patients were receiving combination pharmacotherapy (39.2%), most commonly involving SNRIs and anticonvulsants. This pattern reflects the need for individualized treatment approaches and highlights the challenges of managing fibromyalgia with a single pharmacological agent.

Pain and sleep disturbances are among the most common and burdensome symptoms in patients with fibromyalgia, and they often appear to influence each other [19]. Current treatment approaches vary across guidelines. For example, EULAR (European League Against Rheumatism) recommendations include medications such as pregabalin, cyclobenzaprine, and amitriptyline, alongside non-pharmacological options like meditation, whereas NICE (National Institute of Health and Care Excellence) guidelines take a more limited approach, primarily recommending amitriptyline [5,20]. More recently, interventions such as cognitive-behavioral therapy and neuromodulation techniques—particularly transcranial direct current stimulation combined with exercise—have also been shown to improve sleep quality in this patient group [21,22].

Taken together, these findings reflect the complexity of managing sleep disturbances in fibromyalgia and the lack of a clear, unified treatment strategy. In line with this, our results suggest that both pain intensity and sleep disturbance are closely linked to disease burden and quality of life. In particular, higher NRS-11 scores were associated with increased FIQ scores, while worsening sleep quality was similarly related to greater disease impact and lower SF-12 scores.

Previous studies, including that of Schafranski et al., have reported short-term reductions in pain following intravenous lidocaine treatment [23]. Similarly, we observed a reduction in pain intensity at the 1-month follow-up; however, this effect appeared less pronounced by the third month. Improvements in both physical and mental components of quality of life were also observed at 1 and 3 months compared with baseline.

Lidocaine blocks sodium channels and N-methyl-D-aspartate (NMDA) receptors in the central nervous system and reduces substance P in the dorsal root ganglion. Thus, neuronal hyperexcitability decreases, and symptoms such as allodynia and hyperalgesia subside. Intravenous lidocaine may have potential therapeutic effects in the management of FM and other conditions associated with neuropathic pain; however, its clinical benefits in this population require confirmation in controlled studies [24].

Intravenous local anesthetic therapies have been used for many years, particularly in the treatment of chronic neuropathic pain. Their use has been explored in conditions such as complex regional pain syndrome, diabetic neuropathy, post-stroke pain, and various peripheral neuropathies. In fibromyalgia, symptoms like numbness, tingling, allodynia, and hyperalgesia are also commonly encountered [25].

Previous studies suggest that pharmacological treatments, including anticonvulsants and serotonin–norepinephrine reuptake inhibitors (SNRIs), may offer short- to mid-term improvements in sleep and quality of life. However, these benefits do not always persist, and treatment responses can vary even when medications are used at appropriate doses and durations [26]. In our own clinical experience, some patients use these medications irregularly or discontinue them due to side effects. Symptoms such as gastrointestinal discomfort, dry mouth, dizziness, and concerns about dependence seem to play a role in this. To reduce potential confounding and maintain a more consistent study population, we excluded patients with prior non-adherence or changes in medication during the treatment period.

There is still no clear agreement on the optimal dosing of intravenous lidocaine in fibromyalgia. Different studies have used a wide range of doses, often in combination with other treatments. For example, Wilderman et al. reported that higher and repeated doses of IV lidocaine (5–7.5 mg/kg), administered alongside magnesium, were associated with more sustained effects [27]. Similarly, other studies have used doses between 2 and 7.5 mg/kg, with varying treatment intervals [28].

In our study, IV lidocaine was administered at a dose of 5 mg/kg once weekly, in addition to ongoing medical treatment. This approach was associated with noticeable short-term improvements in sleep quality and quality of life. While PSQI scores improved markedly at 1 month, this effect appeared to diminish by the third month, suggesting that the benefit may be time-limited.

It is widely accepted that a PSQI score of 5 or higher indicates impaired sleep quality [11]. Sleep disturbances are highly prevalent in patients with fibromyalgia, with Andrade et al. reporting that nearly 90% of patients experience concomitant sleep problems [29]. In line with this, patients with sleep disturbances were included in our study, and a cut-off value of 5 was used based on existing literature.

Following intravenous lidocaine treatment, the mean PSQI score decreased from 15.7 at baseline to 5.84 at 1 month and 7.82 at 3 months, suggesting an overall improvement in sleep quality. However, this improvement was not sustained to the same extent over time. By the third month, only a relatively small proportion of patients (21.6%) had PSQI scores within the normal range, while the majority (78.4%) continued to report poor sleep quality.

Taken together, these findings suggest that although intravenous lidocaine may offer some short-term benefit in sleep, its effects appear to diminish over time, and sleep disturbances persist in a substantial proportion of patients.

Emerging evidence points to a role for molecular and genetic factors in the pathophysiology of fibromyalgia. Mitochondrial RNA, along with various genetic and epigenetic mechanisms, has been implicated in disease processes, and metabolites such as 5-hydroxyindoleacetic acid (5-HIAA) have been explored as potential markers of disease activity [30,31,32,33]. Integrating clinical outcomes with such biomarkers in future research may provide a basis for distinguishing patient populations with differential treatment responsiveness to specific treatments, including intravenous lidocaine.

Many studies on intravenous lidocaine in fibromyalgia have primarily focused on pain-related outcomes, with comparatively less attention given to sleep and its interaction with other clinical domains [23,24]. In our study, a more integrated pattern emerged. At 1 month, sleep quality was closely associated with both physical and mental aspects of quality of life, as well as overall disease impact. By 3 months, however, pain intensity appeared to play a more central role in shaping clinical outcomes.

Overall, these findings indicate that the relative contribution of symptom domains may change over time. Sleep seems to be more relevant in the early phase following treatment, whereas pain becomes more influential during longer follow-up. Clinically, this pattern supports the idea that intravenous lidocaine may offer short-term benefits in selected patients, while longer-term effects may require repeated or sustained treatment strategies. These observations also highlight the need for well-designed prospective randomized controlled trials to better define treatment durability and optimize its use in practice.

This study has several limitations that should be kept in mind when interpreting the findings. First, the retrospective design and the absence of a control group make it challenging to establish causality and to clearly distinguish treatment effects from natural variations over time or regression to the mean. Additionally, alternative explanations such as placebo effects and the natural fluctuation of fibromyalgia symptoms cannot be excluded. Second, the relatively small sample size and single-center setting may restrict the generalizability of the results. Third, although concomitant pharmacological treatments were required to remain unchanged, residual confounding related to prior treatments and non-pharmacological interventions cannot be fully excluded. Fourth, sleep quality was assessed exclusively using a self-reported questionnaire, and the lack of objective sleep measurements, such as polysomnography, may limit the precision of sleep-related outcome assessment. Finally, a fixed intravenous lidocaine dosing regimen was used, and the absence of dose-comparison or dose–response analyses precludes conclusions regarding optimal dosing strategies.

## 5. Conclusions

Intravenous lidocaine was associated with short-term improvements in sleep quality and health-related quality of life in patients with fibromyalgia, although these benefits appeared to diminish by the third month. Sleep quality seemed to be more closely related to early functional outcomes, whereas pain intensity emerged as a more prominent factor in mid-term clinical status.

Taken together, these findings suggest that the clinical response to intravenous lidocaine may vary over time and across symptom domains. While some patients may experience meaningful short-term benefit, sustaining these effects may require further optimization of treatment strategies. Future well-designed, large-scale randomized controlled trials are needed to better define the role of intravenous lidocaine in fibromyalgia and to determine optimal dosing and administration approaches.

## Figures and Tables

**Table 1:** Demographic data.

Variables	*N*	Statistics
Gender, *n* (%)	51	
Female		47 (92.2)
Male		4 (7.8)
Age, (year)	51	41.6 ± 9.5
		43 (22–56)
BMI, (kg/m^2^)	51	27.1 ± 3.8
		26.6 (19–35)
Duration of pain, (year)	51	4.3 ± 3.9
		3 (1–21)
Comorbidity, *n* (%)	51	
Present		7 (13.7)
Absent		44 (86.3)
Medical treatment for fibromyalgia, *n* (%)	51	
SNRI		17 (33.3)
Anticonvulsant		12 (23.6)
Non-SNRI antidepressants		2 (3.9)
Combination therapy		20 (39.2)

*N*: Total number of patients; *n*: Number of patients. Quantitative data were displayed as mean ± standard deviation and median (minimum-maximum) values. SNRI: Serotonin–norepinephrine reuptake inhibitor.

**Table 2:** The pre-IV lidocaine treatment, post-treatment 1-month and post-treatment 3-month NRS-11, FIQ, SF-12 and PSQI scores of the patients.

		Mean (S.D.)	Median (Min–Max)	Test Statistics *	*p*
NRS-11	pre	8.08 (0.98)	8 (6–10) ^a^	85.87	<0.001
	1 month	3.80 (1.91)	4 (1–8) ^b^		
	3 month	5.76 (1.56)	6 (3–9) ^c^		
Fibromyalgia Impact Questionnaire	pre	66.68 (8.92)	69 (50–88) ^a^	77.76	<0.001
	1 month	40.63 (9.41)	42 (15–60) ^b^		
	3 month	54.53 (11.15)	54 (30–79) ^c^		
SF-12 Mental Component Score	Pre	27.49 (3.48)	28 (20–33) ^a^	79.03	<0.001
	1 month	40.80 (5.70)	42 (32–52) ^b^		
	3 month	32.02 (4.50)	32 (24–44) ^c^		
SF-12 Physical Component Score	Pre	27.51 (3.71)	28 (20–34) ^a^	91.07	<0.001
	1 month	40.14 (4.37)	42 (32–48) ^b^		
	3 month	34.10 (3.53)	34 (26–42) ^c^		
Pittsburgh Sleep Quality Index	Pre	15.70 (1.61)	15 (12–19) ^a^	93.59	<0.001
	1 month	5.84 (1.94)	5 (3–9) ^b^		
	3 month	7.82 (2.80)	7 (3–15) ^c^		

* Friedman test statistic, ^a–c^: Values sharing the same letter within each column do not differ significantly.

**Table 3:** NRS-11 responder analysis.

	*n* (%)
Worsened	2 (3.9)
Non-responder	29 (56.9)
≥30% responder	12 (23.5)
≥50% responder	8 (15.7)

NRS-11: Numerical Rating Scale; *n*: Number of patients.

**Table 4:** PSQI status at 3 months.

	*n* (%)
Good sleep (≤5)	11 (21.6)
Poor sleep (>5)	40 (78.4)

PSQI: Pittsburgh Sleep Quality Index; *n*: Number of patients.

**Table 5:** Analysis of the correlations between the scales (t0–t1 change—1 month).

	NRS-11		SF-12 Physical Component Scale		SF-12 Mental Component Scale		Fibromyalgia Impact Questionnaire	
t0–t1 Change	r	*p*	r	*p*	r	*p*	r	*p*
NRS-11								
SF-12 Physical Component Scale	−0.870	<0.001 ^x^						
SF-12 Mental Component Scale	−0.915	<0.001 ^x^	0.891	<0.001 ^x^				
Fibromyalgia Impact Questionnaire	0.786	<0.001 ^x^	−0.707	<0.001 ^x^	−0.758	<0.001 ^x^		
Pittsburgh Sleep Quality Index	0.724	<0.001 ^y^	−0.527	<0.001 ^y^	−0.611	<0.001 ^y^	0.560	<0.001 ^y^

^x^ Pearson correlation; ^y^ Spearman’s rho correlation.

**Table 6:** Analysis of the correlations between the scales (t0–t3 change—3 months).

	NRS-11		SF-12 Physical Component Scale		SF-12 Mental Component Scale		Fibromyalgia Impact Questionnaire	
t0–t3 Change	r	*p*	r	*p*	r	*p*	r	*p*
NRS-11								
SF-12 Physical Component Scale	−0.816	<0.001 ^y^						
SF-12 Mental Component Scale	−0.570	<0.001 ^y^	0.659	<0.001 ^y^				
Fibromyalgia Impact Questionnaire	0.857	<0.001 ^y^	−0.783	<0.001 ^y^	−0.590	<0.001 ^y^		
Pittsburgh Sleep Quality Index	0.539	<0.001 ^y^	−0.407	0.003 ^y^	−0.393	0.004 ^y^	0.434	<0.001 ^y^

^y^ Spearman’s rho correlation.

**Table 7:** Path analysis results regarding scales (t0–t1 change).

Dependent Variable	Independent Variable	β_0_	β_1_	Standard Error	Test Statistics	*p*	R^2^
SF-12 Physical Component Scale	← Pittsburgh Sleep Quality Index	−0.478	−1.090	0.283	−3.851	<0.001	0.229
SF-12 Mental Component Scale	← Pittsburgh Sleep Quality Index	−0.612	−1.709	0.312	−5.472	<0.001	0.375
Fibromyalgia Impact Questionnaire	← Pittsburgh Sleep Quality Index	0.571	2.758	0.561	4.917	<0.001	0.326

β_0_: Standardized Beta coefficient; β_1_: Unstandardized Beta coefficient.

**Table 8:** Path analysis results regarding scales (t0–t3 change).

Dependent Variable	Independent Variable	β_0_	β_1_	Standard Error	Test Statistics	*p*	R^2^
SF-12 Physical Component Scale	← Pittsburgh Sleep Quality Index	0.086	0.134	0.140	0.956	0.339	0.720
SF-12 Physical Component Scale	← NRS-11	−0.894	−2.539	0.256	−9.905	<0.001	
SF-12 Mental Component Scale	← Pittsburgh Sleep Quality Index	−0.010	−0.017	0.212	−0.082	0.935	0.484
SF-12 Mental Component Scale	← NRS-11	−0.690	−2.177	0.387	−5.622	<0.001	
Fibromyalgia Impact Questionnaire	← Pittsburgh Sleep Quality Index	−0.010	−0.039	0.300	−0.129	0.897	0.797
Fibromyalgia Impact Questionnaire	← NRS-11	−0.898	6.394	0.548	11.660	<0.001	

## Data Availability

The datasets used and/or analysed during the current study available from the corresponding author on reasonable request.

## Supplementary Materials

The following supporting information can be downloaded at https://www.mdpi.com/article/10.3390/jcm15082887/s1, The STROBE reporting checklist [12,13].

## Abbreviations

The following abbreviations are used in this manuscript:

FM	Fibromyalgia
ACR	American College of Rheumatology
FIQ	Fibromyalgia Impact Questionnaire
NRS-11	Numerical Rating Scale
SF-12	Short Form-12
MCS-12	Mental Component Scale-12
PCS-12	Physical Component Scale-12
PSQI	Pittsburgh Sleep Quality Index
