# Supplementary material for: The Effect of Intravenous Lidocaine Treatment on Sleep and Quality of Life in Fibromyalgia: An Observational Study
Source: J Clin Med. 2026 Apr 10;15(8):2887. doi: 10.3390/jcm15082887 (PMC13116491; doi:10.3390/jcm15082887)
Supplement: Supplementary file 1 [file jcm-15-02887-s001.zip › jcm-4195284-supplementary.pdf]

The STROBE reporting checklist

For checking that observational epidemiology research articles can be understood and used by everyone

How to use this reporting checklist

This reporting checklist allows authors to demonstrate that their manuscripts adhere to the STROBE reporting guideline.

If you have not used a reporting guideline before, read about how and why to use them and check whether STROBE is the most applicable reporting guideline for your work.

Reporting guidelines are most useful when used early in research. When writing a manuscript or application, consider using the full guidance where you'll find explanations and examples for each item.

After writing, demonstrate adherence by completing this checklist:

1. Specify where each item is described (see Note 1).
2. Cite this checklist (See Note 2).
3. Include your completed checklist as a supplement when submitting to a journal so that future readers can use it to find information.

	Item Description	Location (or reason for not reporting)
**Title and abstract**		
1a. Indicate the study's design	Indicate the study's design with a commonly used term in the title or the abstract.	The study design was clearly stated as a retrospective observational study in both the title and the abstract.
1b. Abstract	Provide in the abstract an informative and balanced summary of what was done and what was found.	The abstract provides a clear, informative, and balanced summary of the study, including the objectives, study design, patient population, outcome measures, key results, and conclusions
**Introduction**		
2. Background / rationale	Explain the scientific background and rationale for the investigation being reported.	The scientific background and rationale are clearly described in the Introduction, including the clinical relevance of fibromyalgia, the role of sleep and quality of life, the potential

		effects of intravenous lidocaine, and the existing gap in the literature.
3. Objectives	State specific objectives, including any prespecified hypotheses.	The study objective was clearly defined; however, no explicit prespecified hypotheses were reported
**Methods**		
4. Study design	Present key elements of study design early in the paper.	The study design was clearly specified early in the paper, being described as a retrospective observational cohort study in the Methods section and reflected in the abstract
5. Setting	Describe the setting, locations, and relevant dates, including periods of recruitment, exposure, follow-up, and data collection.	The study setting, location, and time frame were clearly defined, including the tertiary care center where the study was conducted, the recruitment period (June 2023 to June 2024), and the follow-up assessments at baseline, 1 month, and 3 months
6a. Eligibility criteria	**Cohort study:** Give the eligibility criteria, and the sources and methods of selection of participants. Describe methods of follow-up. **Case-control study:** Give the eligibility criteria, and the sources and methods of case ascertainment and control selection. Give the rationale for the choice of cases and controls. **Cross-sectional study:** Give the eligibility criteria, and the sources and methods of selection of participants.	The eligibility criteria, participant selection process, and follow-up methods were clearly described. Participants were retrospectively identified from medical records based on predefined inclusion and exclusion criteria. Follow-up evaluations were performed at baseline, 1 month, and 3 months
6b. Matching criteria	**Cohort study:** For matched studies, give matching criteria and number of exposed and unexposed. **Case-control study:** For matched studies, give matching criteria and the number of controls per case.	Not applicable. This was not a matched study
7. Variables	Clearly define all outcomes, exposures, predictors, potential confounders, and effect modifiers. Give diagnostic criteria, if applicable.	All relevant variables were clearly defined, including exposure (intravenous lidocaine treatment), outcomes (NRS-11, FIQ, SF-12, and PSQI), and diagnostic criteria based on the 2016 ACR guidelines. Potential confounders were

		minimized through strict inclusion and exclusion criteria
8. Data sources / measurement	For each variable of interest give sources of data and details of methods of assessment (measurement). Describe comparability of assessment methods if there is more than one group.	Data sources and measurement methods were clearly described. Clinical variables were obtained from medical records and assessed using standardized and validated instruments (NRS-11, FIQ, SF-12, and PSQI). As the study involved a single cohort, comparability between groups was not applicable
9. Bias	Describe any efforts to address potential sources of bias.	Several measures were taken to address potential sources of bias, including strict eligibility criteria to reduce selection bias, exclusion of patients with missing data, control of treatment-related confounding by excluding patients with treatment changes, and the use of validated instruments to minimize measurement bias.
10. Study size	Explain how the study size was arrived at.	A priori sample size calculation was performed using G*Power (version 3.1.9.4). Assuming an effect size of 0.5, a statistical power of 0.95, and a significance level of 0.05 for non-parametric analysis, the minimum required sample size was estimated to be 47. A total of 51 patients were included in the study, exceeding the required sample size."
11. Quantitative variables	Explain how quantitative variables were handled in the analyses. If applicable, describe which groupings were chosen, and why.	Quantitative variables were handled as continuous measures and assessed for normality using the Kolmogorov–Smirnov test. Statistical analyses were conducted using appropriate parametric or non-parametric methods based on data distribution. No arbitrary categorization of continuous

		variables was applied.
12a. Statistical methods	Describe all statistical methods, including those used to control for confounding.	All statistical methods were clearly described, including descriptive analyses, normality testing (Kolmogorov–Smirnov), non-parametric repeated measures analysis (Friedman test with Bonferroni-adjusted Wilcoxon tests), correlation analyses (Pearson and Spearman), and path analysis. Confounding was primarily controlled at the design stage through predefined inclusion and exclusion criteria. In addition, responder analysis (≥30% and ≥50% reduction in NRS-11) and threshold-based analysis of PSQI (>5) were performed to enhance clinical interpretability.
12b. Statistical methods – subgroups and interactions	Describe any methods used to examine subgroups and interactions.	No formal subgroup or interaction analyses were conducted; however, relationships between variables were explored using correlation and path analysis.
12c. Statistical methods – missing data	Explain how missing data were addressed.	Missing data were addressed through complete-case analysis, with patients who had incomplete clinical data excluded from the study.
12di. Statistical methods – loss to follow-up	**Cohort study:** If applicable, describe how loss to follow-up was addressed.	Loss to follow-up was addressed by restricting the analysis to patients with complete follow-up data at all predefined time points (baseline, 1 month, and 3 months).
12dii. Statistical methods – matching cases and controls	**Case-control study:** If applicable, explain how matching of cases and controls was addressed.	Not applicable. This was not a case-control study.
12diii. Statistical methods – sampling strategy	**Cross-sectional study:** If applicable, describe analytical methods taking account of sampling strategy.	Not applicable. This was not a cross-sectional study.
12e. Statistical	Describe any sensitivity analyses.	No sensitivity analyses were

methods – sensitivity analyses		conducted.
**Results**		
13a. Participant numbers	Report the numbers of individuals at each stage of the study—e.g., numbers potentially eligible, examined for eligibility, confirmed eligible, included in the study, completing follow-up, and analysed; Consider use of a flow diagram.	The number of participants at each stage of the study was clearly reported, including records reviewed for eligibility (n = 281), excluded patients (n = 230), and those included in the final analysis (n = 51). A flow diagram was used to illustrate the selection process
13b. Participants – non-participation	Give reasons for non-participation at each stage.	Reasons for non-participation were clearly reported, including not meeting inclusion criteria (n = 183), declined treatment (n = 23), and missing clinical data (n = 24).
13c. Participants – flow diagram	Consider use of a flow diagram.	A flow diagram was used to present the participant selection process and reasons for exclusion at each stage
14a. Descriptive data – participant characteristics	Give characteristics of study participants (e.g., demographic, clinical, social) and information on exposures and potential confounders. Present the information in a table.	Participant characteristics, including demographic and clinical variables, were summarized in Table 1. Exposure to intravenous lidocaine was consistent across all participants. Potential confounders were minimized through predefined inclusion and exclusion criteria.
14b. Descriptive data – missing data	Indicate the number of participants with missing data for each variable of interest.	Patients with incomplete clinical data were excluded; however, the extent of missing data for each variable was not reported individually.
14c. Descriptive data – follow-up time	**Cohort study:** Summarise follow-up time—e.g., average and total amount.	Participants were followed at baseline, 1 month, and 3 months after treatment; however, overall summary measures of follow-up time were not explicitly calculated. The total follow-up duration for each participant was 3 months.

15. Outcome data	**Cohort study:** Report numbers of outcome events or summary measures over time. **Case-control study:** Report numbers in each exposure category, or summary measures of exposure. **Cross-sectional study:** Report numbers of outcome events or summary measures.	Outcome measures were reported longitudinally, with summary statistics (mean, standard deviation, median, and range) presented for NRS-11, FIQ, SF-12, and PSQI scores at baseline, 1 month, and 3 months.
16a. Main results	Give unadjusted estimates and, if applicable, confounder-adjusted estimates and their precision (e.g., 95% confidence intervals). Make clear which confounders were adjusted for and why they were included.	In addition to unadjusted analyses, clinically interpretable measures were incorporated, including responder analysis (≥30% and ≥50% reduction in NRS-11) and threshold-based evaluation of PSQI (>5). These complementary analyses provide a more clinically meaningful interpretation of the findings beyond p-values alone.
16b. Main results – category boundaries	Report category boundaries when continuous variables were categorised.	Not applicable. Continuous variables were not categorized.
16c. Main results – risk	If relevant, consider translating estimates of relative risk into absolute risk for a meaningful time period.	Not applicable, as the study focused on changes in continuous outcome measures rather than relative risk estimates.
17. Other analyses	Report other analyses done—e.g., analyses of subgroups and interactions, and sensitivity analyses.	Additional analyses included correlation and path analysis, as well as clinically oriented analyses such as responder analysis based on NRS-11 and threshold-based evaluation of PSQI at 3 months.
**Discussion**		
18. Key results	Summarise key results with reference to study objectives.	The key findings were clearly summarized in relation to the study objective, showing that intravenous lidocaine treatment was associated with significant improvements in pain, sleep quality, and quality of life, with the greatest effects observed at 1 month and partial attenuation at 3 months

19. Limitations	Discuss limitations of the study, taking into account sources of potential bias or imprecision. Discuss both direction and magnitude of any potential bias.	The limitations of the study were clearly discussed, including potential sources of bias such as the retrospective design, absence of a control group, limited sample size, and single-center setting. While these factors may have influenced the results, the direction and magnitude of potential biases were not explicitly assessed. Furthermore, clinical interpretation was supported by additional analyses; however, the absence of universally accepted MCID thresholds for some scales may limit the generalizability of these interpretations.
20. Interpretation	Give a cautious overall interpretation considering objectives, limitations, multiplicity of analyses, results from similar studies, and other relevant evidence.	The results were interpreted cautiously in light of the study objectives, methodological limitations, and findings from previous studies, with appropriate emphasis on the need for further randomized controlled trials to confirm the results. The interpretation was further strengthened by distinguishing statistical significance from clinical relevance through additional analyses.
21. Generalisability	Discuss the generalisability (external validity) of the study results.	The generalisability of the findings may be limited due to the single-center design, relatively small sample size, and the inclusion of patients from a tertiary care pain clinic, which may not fully represent the broader fibromyalgia population.
**Other information**		
22. Funding	Give the source of funding and the role of the funders for the present study and, if applicable, for the original study on which the present article is based.	The study received no external funding. Therefore, no funders had any role in the study

		design, data collection, analysis, interpretation, or manuscript preparation.

1 How to specify where content is

Tell the reader where they can find information. E.g.,

- Results; paragraph 2
- Methods, Participants; paragraphs 1 & 2.
- Table 3
- Supplement B, para. 4

If you have chosen not to describe an item, explain why. You can do this in the checklist, or as a note below it.

You can describe items in the article body, or in tables, figures, or supplementary materials, and should prioritize items you feel are most important to your intended audience. The order of items in your manuscript does not need to match the order of items in this checklist. You can decide how best to structure your work.

2 How to cite

Describe how you used STROBE at the end of your Methods section, referencing the resources you used e.g.,

> ‘We used the STROBE reporting guideline(1) to draft this manuscript, and the STROBE reporting checklist(2) when editing, included in supplement A’

If you use a reporting checklist, remember to include it as a supplement when publishing so that readers can easily find information and see how you have interpreted the guidance.
